# Excess US Firearm Mortality During the COVID-19 Pandemic Stratified by Intent and Urbanization

**DOI:** 10.1001/jamanetworkopen.2023.23392

**Published:** 2023-07-13

**Authors:** Eric W. Lundstrom, Caroline P. Groth, James E. Harrison, Brian Hendricks, Gordon S. Smith

**Affiliations:** 1 Department of Epidemiology and Biostatistics, School of Public Health, West Virginia University, Morgantown; 2 College of Medicine and Public Health, Flinders University, Adelaide, South Australia

## Abstract

This cross-sectional study used time series forecasting to estimate excess firearm mortality in the US during the COVID-19 pandemic.

## Introduction

Published research^1,2^ suggests the COVID-19 pandemic exacerbated already increasing rates of overall US firearm mortality. Descriptive comparisons of 2019 to 2020 mortality data suggest this early trend was driven by homicides and urban firearm deaths.^2^ However, to our knowledge, no study has quantified excess intent- and urbanization-stratified US firearm deaths throughout the pandemic using inferential statistical methods. We estimate overall and intent- and urbanization-stratified excess US firearm mortality throughout the COVID-19 pandemic.

## Methods

In this cross-sectional study, monthly US firearm mortality data for 1999 to 2021 (most recent) were extracted from CDC WONDER.^3^ Data on race and ethnicity were not collected because they were not used in the hierarchical time series modeling or forecasting approach. CDC WONDER also does not report mean age. Urbanization- and intent-stratified mortality were aggregated using a hierarchical time series structure, with urbanization- and intent-stratified deaths aggregated to intent level and intent aggregates combined into a final aggregate time series. Urbanization-level forecasts were produced by aggregating urbanization- and intent-stratified deaths to the urbanization level. The West Virginia University Institutional Review Board deemed this study exempt because it did not meet the definition of human subjects research. This study followed the STROBE reporting guideline.

A counterfactual scenario was created in which US firearm mortality continued prepandemic trends. Counterfactual mortality estimates were created by forecasting 21 months of pandemic-era (April 2020 to December 2021) data using an ensemble time series model of prepandemic (January 1999 to March 2020) data. This approach combines forecasts of multiple time series models and has improved accuracy over single-model approaches.^4^ Our ensemble model pooled autoregressive integrated moving average and exponential smoothing forecasts using inverse variance weighting. Minimum trace forecast reconciliation was used to integrate the unique influence of each hierarchical time series level into final aggregate forecasts.

Statistical analyses were performed in RStudio, version 4.2.2 (R Foundation); 2-sided hypotheses were tested at α = .01. Detailed methods, including data and RStudio code/packages used to produce and validate forecasts, are available in the eAppendix in Supplement 1.

## Results

During the pandemic, 83 966 firearm deaths occurred vs 73 194 predicted deaths, representing a 14.7% increase above expected (99% prediction interval [PI], 13.6%-15.9%) (Table). Stratified by intent, firearm homicides showed the greatest increase above expected values (9329 additional deaths [34.1%; 99% PI, 31.4%-36.8%]), followed by other (327 deaths [13.2%; 99% PI, 9.0%-17.7%]) and suicide (1116 deaths [2.6%; 99% PI, 1.2%-4.0%]). Larger increases were observed in urban areas, and homicides showed the largest percentage increase for each urbanization category. Monthly homicide firearm deaths were higher than forecasted estimates throughout the pandemic (Figure); firearm suicides were not higher than forecasted until later in the pandemic.

**Table.** Observed and Forecasted Firearm Deaths During the COVID-19 Pandemic (April 2020 to December 2021), Both Overall and Stratified by Intent and Urbanization^a^

Category	Actual No. of deaths	Total forecasted deaths (99% prediction interval)	Change from expected (99% prediction interval), %
Total	83 966	73 196 (72 459 to 73 933)	14.7 (13.6 to 15.9)
Homicide^b^	36 723	27 394 (26 835 to 27 952)	34.1 (31.4 to 36.8)
Large central metropolitan area	16 471	12 001 (11 459 to 12 544)	37.2 (31.3 to 43.7)
Large fringe metropolitan area	6259	4560 (4324 to 4797)	37.3 (30.5 to 44.8)
Medium metropolitan area	7565	5916 (5662 to 6169)	27.9 (22.6 to 33.6)
Micropolitan (nonmetropolitan) area	2307	1724 (1576 to 1872)	33.8 (23.3 to 46.4)
Noncore (nonmetropolitan) area	1573	1178 (1055 to 1302)	33.5 (20.8 to 49.1)
Small metropolitan area	2548	2014 (1888 to 2140)	26.5 (19.0 to 34.9)
Suicide^b^	44 469	43 352 (42 776 to 43 928)	2.6 (1.2 to 4.0)
Large central metropolitan area	9097	8840 (8515 to 9164)	2.9 (−0.7 to 6.8)
Large fringe metropolitan area	9608	9607 (9238 to 9976)	0 (−3.7 to 4.0)
Medium metropolitan area	10 483	9708 (9384 to 10 033)	8.0 (4.5 to 11.7)
Micropolitan (nonmetropolitan) area	5441	5413 (5161 to 5666)	0.5 (−4.0 to 5.4)
Noncore (nonmetropolitan) area	4452	4214 (4039 to 4388)	5.7 (1.4 to 10.2)
Small metropolitan area	5388	5570 (5335 to 5806)	−3.3 (to −7.2 to 1)
Other^b^	2774	2450 (2356 to 2544)	13.2 (9.0 to 17.7)
Large central metropolitan area	684	549 (486 to 612)	24.5 (11.8 to 40.6)
Large fringe metropolitan area	440	380 (332 to 427)	15.9 (3.0 to 32.5)
Medium metropolitan rea	662	589 (527 to 651)	12.5 (1.8 to 25.7)
Micropolitan (nonmetropolitan) area	369	318 (280 to 356)	16.1 (3.6 to 32.0)
Noncore (nonmetropolitan) area	286	280 (248 to 311)	2.3 (−8.1 to 15.4)
Small metropolitan area	334	335 (304 to 367)	−0.4 (−9.1 to 10)
Large central metropolitan area^b^	26 252	21294 (20 814 to 21 776)	23.3 (20.6 to 26.1)
Homicide	16471	11 897 (11 402 to 12 394)	38.4 (32.9 to 44.5)
Suicide	9097	8854 (8536 to 9172)	2.7 (−0.8 to 6.6)
Other	684	543 (476 to 610)	26.0 (12.1 to 43.8)
Large fringe metropolitan area^b^	16 307	14 481 (14 182 to 14 780)	12.6 (10.3 to 15)
Homicide	6259	4514 (4294 to 4734)	38.7 (32.2 to 45.8)
Suicide	9608	9592 (9284 to 9900)	0.2 (−2.9 to 3.5)
Other	440	375 (326 to 424)	17.3 (3.7 to 35)
Medium metropolitan area^b^	18 710	16 286 (16 000 to 16 574)	14.9 (12.9 to 16.9)
Homicide	7565	5923 (5693 to 6154)	27.7 (22.9 to 32.9)
Suicide	10 483	9777 (9501 to 10 053)	7.2 (4.3 to 10.3)
Other	662	586 (521 to 652)	12.9 (1.5 to 27.2)
Small metropolitan area^b^	8270	7956 (7745 to 8168)	3.9 (1.3 to 6.8)
Homicide	2548	2017 (1894 to 2140)	26.3 (19.1 to 34.5)
Suicide	5388	5604 (5406 to 5803)	−3.9 (−7.1 to 0.3)
Other	334	335 (303 to 367)	−0.4 (−9.2 to 10.2)
Micropolitan (nonmetropolitan) area^b^	8117	7489 (7265 to 7691)	8.4 (5.4 to 11.6)
Homicide	2307	1723 (1581 to 1861)	33.9 (23.8 to 45.7)
Suicide	5441	5450 (5235 to 5646)	−0.2 (−3.8 to 3.8)
Other	369	317 (278 to 356)	16.5 (3.7 to 32.9)
Noncore (nonmetropolitan) area^b^	6311	5758 (5594 to 5922)	9.6 (6.6 to 12.8)
Homicide	1573	1193 (1078 to 1308)	31.9 (20.3 to 45.9)
Suicide	4452	4284 (4134 to 4434)	3.9 (0.4 to 7.7)
Other	286	281 (249 to 313)	1.7 (−8.7 to 14.8)

^a^ Intents were defined as homicide (*International Statistical Classification of Diseases and Related Health Problems, Tenth Revision [ICD-10]* underlying cause of death codes U01.4, X93-X95), suicide (X72-X74), and other. Other intent was defined as any firearm death due to legal intervention or war (Y35.0), unintentional intent (W32-W34), or undetermined intent (Y22-Y24).

^b^ Each primary category represented the forecasted mortality for that category as an hierarchical time series structure incorporating the influence of each category below it via minimum trace forecast reconciliation.

**Figure.**
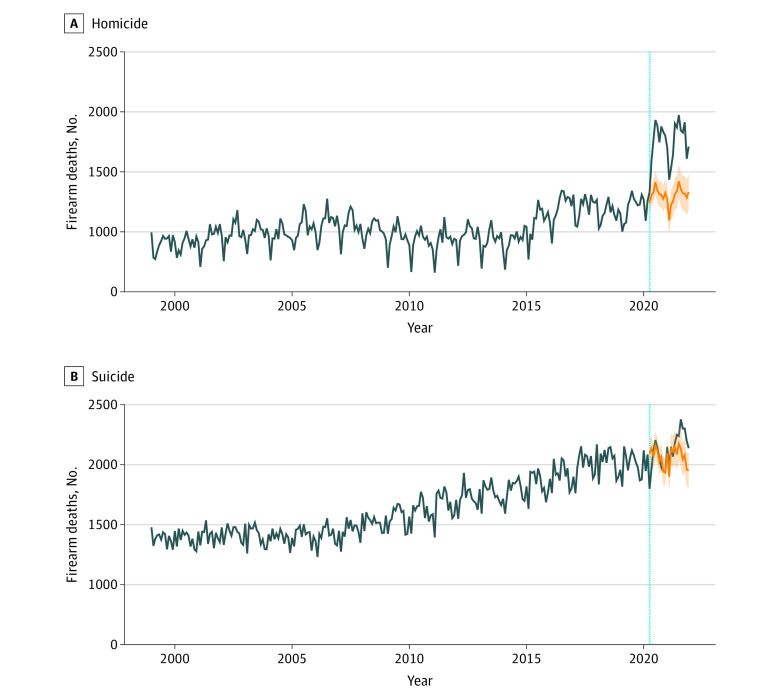
Observed Monthly Firearm Deaths in the US Compared With Counterfactual Forecasts With 99% Prediction Intervals Vertical dotted line indicates the beginning of the COVID-19 pandemic in the US (April 2020), with blue lines from this point indicating observed deaths and orange lines indicating counterfactual forecasts with 99% prediction intervals. Forecasts were created using ensemble autoregressive integrated moving average and exponential smoothing time series model.

## Discussion

Excess firearm mortality during the COVID-19 pandemic was primarily driven by homicides and gun deaths in urban areas. This finding supports descriptive comparisons of 2019 to 2020 firearm deaths^2^ and studies^1,5^ demonstrating that firearm-involved violence increased during the first year of the pandemic. We build on these studies using inferential statistical methods, intent and urbanization stratification, and data through 2021.

This study used ensemble time series forecasting, which enhances accuracy over single-model approaches^4^ and improves on methods established in previous pandemic-era counterfactual forecasting studies.^5,6^ However, our counterfactual scenario represents a forecast based on historical trends and did not include data potentially influencing prepandemic firearm mortality, such as firearm ownership rates. Additionally, we used count not rate data. Nonetheless, this approach is consistent with previous studies of excess injury mortality using US vital statistics data,^6^ which do not provide subannual population-adjusted rates.^3^

Notwithstanding its limitations, our study found that most excess firearm mortality during the COVID-19 pandemic was attributable to homicide and deaths in urban areas. Future research should study stratification by sex, race, and gun type (eg, handgun) and elucidate any relationship our findings have with excess firearm purchasing observed early in the pandemic.^5^

## Notes

Supplement 1. **eAppendix.** Detailed Methodology

Supplement 2.
Data Sharing Statement
