# Supplementary material for: Excess US Firearm Mortality During the COVID-19 Pandemic Stratified by Intent and Urbanization
Source: JAMA Netw Open. 2023 Jul 13;6(7):e2323392. doi: 10.1001/jamanetworkopen.2023.23392 (PMC10346122; doi:10.1001/jamanetworkopen.2023.23392)
Supplement: Supplement 1. — eAppendix. Detailed Methodology [file jamanetwopen-e2323392-s001.pdf]

Supplementary Online Content

Lundstrom EW, Groth CP, Harrison JE, Hendricks B, Smith GS. Excess US firearm mortality during the COVID-19 pandemic stratified by intent and urbanization. *JAMA Netw Open*. 2023;6(7):e2323392. doi:10.1001/jamanetworkopen.2023.23392

eAppendix. Detailed Methodology

This supplementary material has been provided by the authors to give readers additional information about their work.

© 2023 Lundstrom EW et al. *JAMA Network Open.*

eAppendix. Detailed Methodology

Analyses were performed using RStudio version 4.2.2.[1] The RStudio packages used in this study were ‘anytime’,[2] ‘distributional’,[3] ‘fable’,[4] ‘fable.tools’,[5] ‘forecast’,[6] ‘imputeTS’,[7] ‘progressr’,[8] ‘readxl’,[9] ‘tidyverse’,[10] ‘tsibble’,[11] and ‘zoo’.[12] The RStudio code used to generate and validate forecasts is available at the end of this document.

Data extraction

Monthly firearm mortality data for 1999-2021 were extracted from the Centers for Disease Control and Prevention Wide-ranging Online Data for Epidemiologic Research (CDC WONDER) database.[13] CDC WONDER collects and compiles mortality data from state-level death certificate registries. Firearm deaths were defined as those with a firearm-involved ICD-10 underlying cause of death (UCOD) code. Monthly mortality was stratified by intent and urbanization. Intents were defined as homicide (ICD-10 UCOD codes U01.4, X93–X95), suicide (X72–X74), and other; the other intent category was comprised of firearm deaths due to unintentional (W32–W34), undetermined (Y22–Y24), and legal intervention or war (Y35.0) firearm deaths. Urbanization strata were based on 2013 urbanization codes; from most to least urban, these are large central metro, large fringe metro, medium metro, small metro, micropolitan (nonmetro), and noncore (nonmetro).

Missing data imputation

Monthly mortality counts for firearm deaths attributable to intents other than homicide and suicide were 10.5% missing due to data suppression; CDC WONDER does not report data values less than 10. Missing values were imputed via Kalman filter using the na_kalman() command in the RStudio package ‘impute TS’.[7] Kalman filtering uses an algorithmic smoothing approach to assessing the components of a time series data and is estimated based on maximum likelihood estimation.[14]

Hierarchical ensemble time series forecasting

Following the approach of previous literature,[15–17] we used time series forecasting to create a counterfactual scenario in which firearm injury followed pre-pandemic trends. We used a standard structure where we defined our *training set* to be from January 1999 to March 2020 and our *testing dataset* to include monthly data from April 2020 to December 2021. This structure is described in Figure S1. Using just the training dataset, we developed a time series model reflecting over-time trends in firearm fatalities. Then using this model, we predicted (or forecasted) firearm fatalities monthly for the months of the test dataset (i.e., April 2020-December 2021). For each prediction, we also developed 99% prediction intervals which creates an uncertainty interval around each predicted value. These forecasted values (known as our *counterfactual forecasted values*) were then compared to the actual measurements observed in April 2020-December 2021.

For modeling we used all test data across the urbanization and intent strata. By placing all strata in the model together, we could also assess trends in the overall firearm fatality rates. We did this by assuming a hierarchical structure with two levels (one level as the overall dataset and another level being each urbanization and intent category).

We applied both autoregressive integrated moving average (ARIMA) and exponential smoothing (ETS) modeling to our training dataset. Briefly, an ARIMA model is specified by the form ARIMA $(p,d,q)(P,D,Q)s$, where p, d, and q are the autoregressive, differencing, and moving average components of the model, respectively; P, D, and Q are seasonal variants of these components; and s is the order of seasonality.[20] ETS models capture error, trend, and seasonality components of a time series, each of which may be additive or multiplicative in nature; thus, accounting for all combinations, a total of 35 ETS model structures are possible.[21]All model selection was performed using the ‘fable’[4] and ‘fabletools’[5] RStudio packages, which perform automatic model selection (p, d, q, P, D, Q, and s for ARIMA models; E, T, and S for ETS models) based on minimization of Akaike information criteria (AIC); model selection via these packages have been used in numerous public health forecasting studies,[23–25] including of injury mortality data.[26]

To incorporate both ARIMA and ETS components into the models generating our counterfactual forecasts, we used ensemble time series modeling. Ensemble time series modeling incorporates components of multiple time series models into a single model to improve predictive accuracy. The predictive benefits of ensemble (as opposed to

single model) forecasting approaches are derived from their inherent ability to capture different patterns present within real-world time series data.[22] In our ensemble model, ARIMA and ETS components were weighted based on the inverse of their variance. This method has the benefit of minimizing the influence of a model component contributing a large error to the forecast and therefore reduces final forecasts' confidence intervals. ARIMA/ETS model parameters were estimated using maximum likelihood method,[4] with fit residuals for the final, aggregate model approximating a normal distribution; Shapiro-Wilks test of fit residuals was non-significant. Ljung-box test was insignificant for the final aggregate model, indicating serial correlation among residuals was random and equal to white noise.[27,28]

Model validation

To assess our ensemble model's predictive ability, and therefore its capacity to generate realistic counterfactual trends, we used time series cross validation (TSCV). Cross validation assesses the accuracy of a given model by sub-setting time series data into training and test sets as described above. TSCV does this cross validation procedure on a rolling basis, meaning that we repeat the cross validation process many times using slightly different training and tests sets.[29] Specifically, we first fit ARIMA/ETS ensemble models to five year (January 1999 to December 2004) of pre-pandemic HTS data and forecasted (predicted) 21 months into the future (January 2005 to September 2006); this was the length of pandemic data that was ultimately forecasted. These 21 months of forecasted data (predictions) were then compared statistically to the real data for the same period. Next, the training window was extended by one month (making it February 1999 to January 2005) and 21 months of data (February 2005 to October 2006) were forecasted and again compared to extant data. This process was continued throughout the entirety of the pre-pandemic period. Using this process, we also compared our ensemble ARIMA/ETS model to non-ensemble ARIMA and ETS model.

TSCV-generated forecasts were compared to real data using mean absolute percent error (MAPE). MAPE is an absolute measure of error divided by the corresponding observed value y:

$$Mean\ absolute\ percent\ error = mean\left(\left|\frac{(y_t - \hat{y}_t) \times 100}{y_t}\right|\right)$$

MAPE is unit-free, always positive, and may be used to compare forecast accuracy across different datasets.[30] Smaller values of MAPE are preferred.

Model validation via TSCV indicated that ARIMA/ETS ensemble models sufficiently forecast pre-pandemic firearm deaths. Averaged across all strata, mean MAPE was lower for ensemble ARIMA/ETS forecasts than for non-ensemble ARIMA or ETS forecasts.

© 2023 Lundstrom EW et al. *JAMA Network Open.*

eReferences

1. RStudio Team. RStudio: Integrated Development for R. Published online February 22, 2022. Accessed March 29, 2022. http://www.rstudio.com/
2. Eddelbuettel D. anytime: Anything to “POSIXct” or “Date” Converter. Published online September 27, 2020. Accessed May 20, 2023. https://cran.r-project.org/web/packages/anytime/index.html
3. O’Hara-Wild M, Kay M, Hayes A, Wang E. distributional: Vectorised Probability Distributions. Published online March 23, 2023. Accessed May 20, 2023. https://cloud.r-project.org/web/packages/distributional/index.html
4. O’Hara-Wild M, Hyndman R, Wang E, Cacers G, Hensel TG, Hyndman T. fable: Forecasting Models for Tidy Time Series. Published online 2021. Accessed May 20, 2023. https://cran.r-project.org/web/packages/fable/index.html
5. O’Hara-Wild M, Hyndman R, Wang E, Cook D, Holt D. fabletools: Core Tools for Packages in the “fable” Framework. Published online April 4, 2023. https://cran.r-project.org/web/packages/fabletools/index.html
6. Hyndman R, Athanasopoulos G, Bergmeir C, et al. forecast: Forecasting Functions for Time Series and Linear Models. Published online February 27, 2023. Accessed May 20, 2023. https://cran.r-project.org/web/packages/forecast/index.html
7. Moritz S, Gatscha S, Wang E, Hause R. imputeTS: Time Series Missing Value Imputation. Published online September 9, 2022. Accessed May 20, 2023. https://cran.r-project.org/web/packages/imputeTS/index.html
8. Bengtsson H. progressr: An Inclusive, Unifying API for Progress Updates. Published online January 10, 2023. Accessed May 20, 2023. https://cran.r-project.org/web/packages/progressr/index.html
9. Wickham H, Bryan J, Kalicinski Ma, et al. readxl: Read Excel Files. Published online February 9, 2023. Accessed May 20, 2023. https://cran.r-project.org/web/packages/readxl/index.html
10. Wickham H, RStudio. tidyverse: Easily Install and Load the “Tidyverse.” Published online February 22, 2023. Accessed May 20, 2023. https://cran.r-project.org/web/packages/tidyverse/index.html
11. Wang E, Cook D, Hyndman R, O’Hara-Wild M, Smith T, Davis W. tsibble: Tidy Temporal Data Frames and Tools. Published online October 9, 2022. Accessed May 20, 2023. https://cran.r-project.org/web/packages/tsibble/index.html
12. Zeileis A, Grothendieck G, Ryan JA, Ulrich JM, Andrews F. zoo: S3 Infrastructure for Regular and Irregular Time Series (Z’s Ordered Observations). Published online April 13, 2023. Accessed May 20, 2023. https://cran.r-project.org/web/packages/zoo/index.html
13. Centers for Disease Control and Prevention. Wide-ranging Online Data for Epidemiologic Research (WONDER). Published September 17, 2022. Accessed October 19, 2022. https://wonder.cdc.gov
14. Afrifa-Yamoah E, Mueller UA, Taylor SM, Fisher AJ. Missing data imputation of high-resolution temporal climate time series data. *Meteorological Applications*. 2020;27(1). doi:10.1002/met.1873
15. Schleimer JP, McCort CD, Shev AB, et al. Firearm purchasing and firearm violence during the coronavirus pandemic in the United States: a cross-sectional study. *Inj Epidemiol*. 2021;8(1). doi:10.1186/s40621-021-00339-5
16. Inada H, Ashraf L, Campbell S. COVID-19 lockdown and fatal motor vehicle collisions due to speed-related traffic violations in Japan: A time-series study. *Injury Prevention*. 2021;27(1):98-100. doi:10.1136/injuryprev-2020-043947
17. Cartus AR, Li Y, Macmadu A, et al. Forecasted and Observed Drug Overdose Deaths in the US during the COVID-19 Pandemic in 2020. *JAMA Netw Open*. 2022;5(3):E223418. doi:10.1001/jamanetworkopen.2022.3418
18. Wickramasuriya SL, Athanasopoulos G, Hyndman RJ. Optimal Forecast Reconciliation for Hierarchical and Grouped Time Series Through Trace Minimization. *J Am Stat Assoc*. 2019;114(526):804-819. doi:10.1080/01621459.2018.1448825

© 2023 Lundstrom EW et al. *JAMA Network Open.*

19. Hyndman R, Athanasopoulos G. Forecast reconciliation. In: *Forecasting: Principles and Practice*. 3rd ed. Otexts; 2021.

20. Hyndman RJ, Athanasopoulos G. Seasonal ARIMA models. In: *Forecasting: Principles and Practice*. 3rd ed. Otexts; 2021.

21. Svetunkov I. ETS Taxonomy. In: *Forecasting and Analytics from ADAM*. ; 2023. Accessed March 19, 2023. https://openforecast.org/adam/ETSConventional.html

22. Wang X, Hyndman RJ, Li F, Kang Y. Forecast combinations: An over 50-year review. *Int J Forecast*. Published online December 2022. doi:10.1016/j.ijforecast.2022.11.005

23. Wang JJJ, Fung T, Weatherburn D. The impact of the COVID-19, social distancing, and movement restrictions on crime in NSW, Australia. *Crime Sci*. 2021;10(1). doi:10.1186/s40163-021-00160-x

24. Boogaerts T, Quireyns M, De Loof H, et al. Do the lockdown-imposed changes in a wastewater treatment plant catchment's socio-demographics impact longitudinal temporal trends in psychoactive pharmaceutical use? *Science of the Total Environment*. 2023;876. doi:10.1016/j.scitotenv.2023.162342

25. Chakma B, Gomes D, Filipe PA, Soares P, de Sousa B, Nunes C. A temporal analysis on patient and health service delays in pulmonary tuberculosis in Portugal: inter and intra-regional differences and in(equalities) between gender and age. *BMC Public Health*. 2022;22(1). doi:10.1186/s12889-022-14216-3

26. Kandula S, Olfson M, Gould MS, Keyes KM, Shaman J. Hindcasts and forecasts of suicide mortality in US: A modeling study. *PLoS Comput Biol*. 2023;19(3):e1010945. doi:10.1371/journal.pcbi.1010945

27. Box GEP, Jenkins GM, Reinsel GC, Ljung GM. *Time Series Analysis: Forecasting and Control*. 5th ed. Wiley; 2016.

28. Ljung GM, Box GEP. On a measure of lack of fit in time series models. *Biometrika*. 1978;65(2):297-303. doi:10.1093/biomet/65.2.297

29. Hyndman R, Athanasopoulos G. Time series cross-validation. In: *Forecasting: Principles and Practice*. 3rd ed. Otexts; 2021. Accessed November 3, 2022. https://otexts.com/fpp3/tscv.html

30. Hyndman RJ, Athanasopoulos G. Evaluating point forecast accuracy. In: *Forecasting: Principles and Practice*. 3rd ed. OTexts; 2021. Accessed October 12, 2022. https://otexts.com/fpp3/accuracy.html

© 2023 Lundstrom EW et al. *JAMA Network Open.*

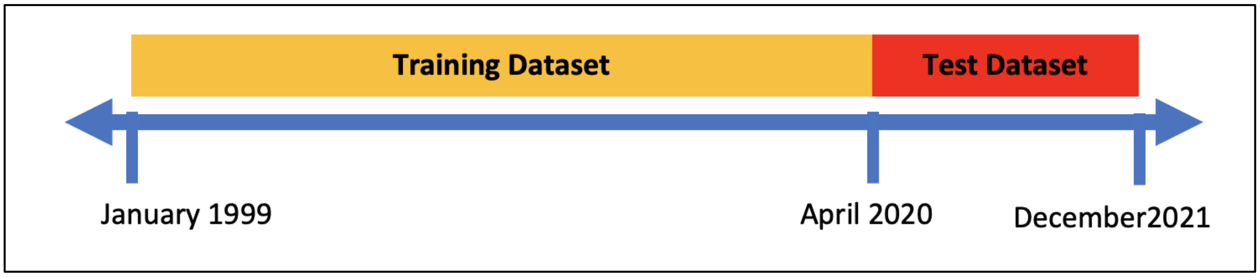

eFigure 1. Depiction of training and test datasets.

© 2023 Lundstrom EW et al. *JAMA Network Open.*

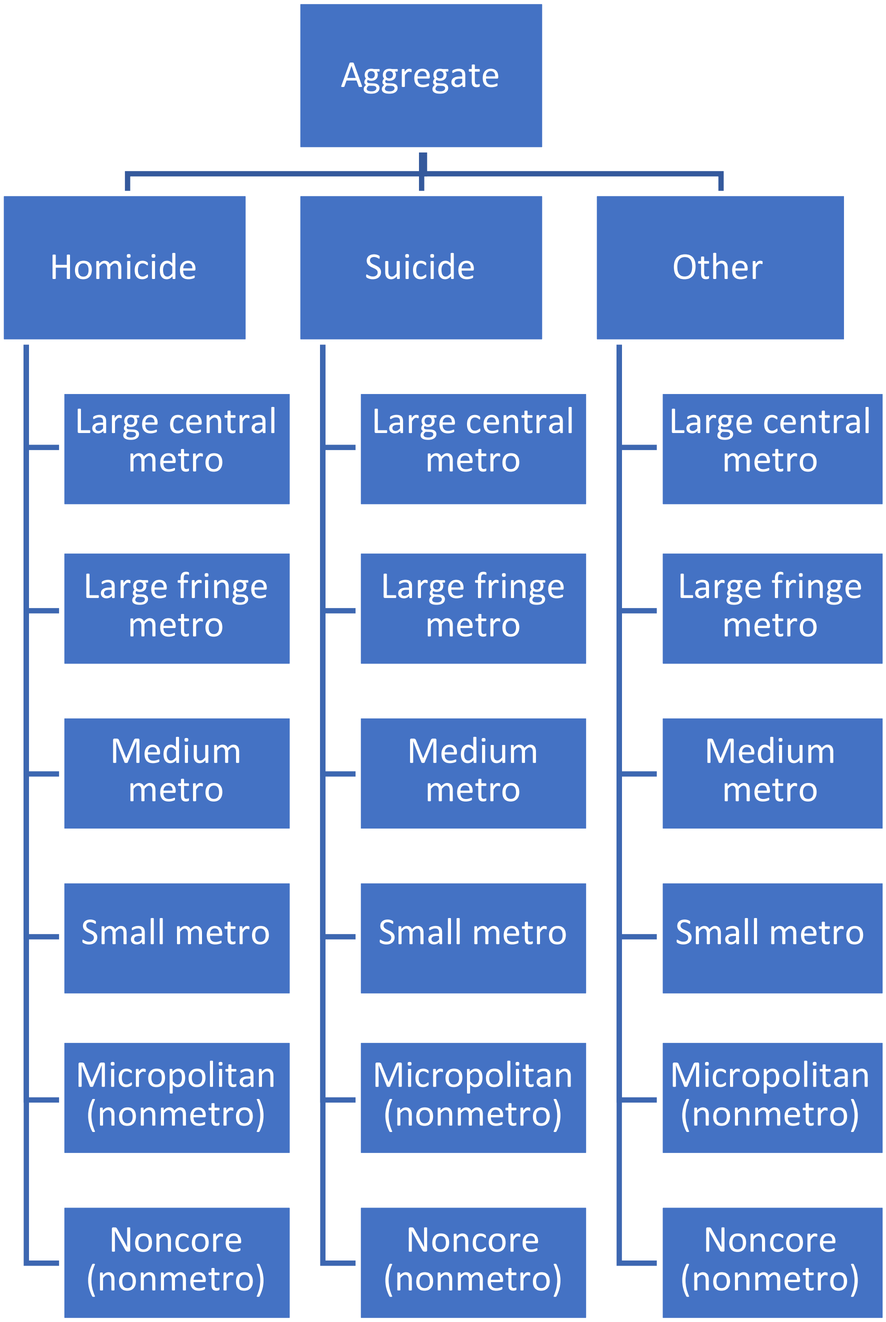

eFigure 2. Hierarchical time series structure used to model total and intent-stratified firearm fatality deaths. The individual mortality of each level is summed to the level above it; deaths stratified by urbanization and intent are summed to the intent level and intent level is summed to total deaths (i.e., "aggregate" in this figure).

© 2023 Lundstrom EW et al. *JAMA Network Open.*

RStudio Code:

```
#### Loading Packages ####
library(tidyverse)
library(tsibble)
library(imputeTS)
library(anytime)
library(forecast)
library(readxl)
library(anytime)
library(zoo)
library(distributional)
library(fable)
library(fabletools)
library(feasts)
library(progressr)

handlers(global = TRUE)

set.seed(100)

FirearmDeaths <- as.data.frame(read_excel("FILE_PATH/USFirearmForecasting_RawData.xlsx"))




#### Imputing Missing Values in "Other" Intent by Urbanization Category ####


###### NonCore (Nonmetro) ######
NonCore_temp <- FirearmDeaths %>% filter(Intent == "Other", Urbanization == "NonCore (Nonmetro)")
NonCore_TS <- ts(NonCore_temp$Deaths, start=c(1999, 1), end=c(2021, 12), frequency=12)
NonCore_TS_Clean <- as_tibble(na_kalman(NonCore_TS))
NonCore_TS_Clean$Urbanization <- "NonCore (Nonmetro)"
NonCore_TS_Clean$Intent <- "Other"
NonCore_TS_Clean$Date <- as.yearmon(seq.Date(from = as.Date("1999-01-01"), to = as.Date("2021-12-021"), by
= "month"))


###### Micropolitan (Nonmetro) ######
Micropolitan_temp <- FirearmDeaths %>% filter(Intent == "Other", Urbanization == "Micropolitan (Nonmetro)")
Micropolitan_TS <- ts(Micropolitan_temp$Deaths, start=c(1999, 1), end=c(2021, 12), frequency=12)
Micropolitan_TS_Clean <- as_tibble(na_kalman(Micropolitan_TS))
Micropolitan_TS_Clean$Urbanization <- "Micropolitan (Nonmetro)"
Micropolitan_TS_Clean$Intent <- "Other"
Micropolitan_TS_Clean$Date <- as.yearmon(seq.Date(from = as.Date("1999-01-01"), to = as.Date("2021-12-021"),
by = "month"))


###### Small Metro ######
SmallMetro_temp <- FirearmDeaths %>% filter(Intent == "Other", Urbanization == "Small Metro")
SmallMetro_TS <- ts(SmallMetro_temp$Deaths, start=c(1999, 1), end=c(2021, 12), frequency=12)
SmallMetro_TS_Clean <- as_tibble(na_kalman(SmallMetro_TS))
```

© 2023 Lundstrom EW et al. *JAMA Network Open.*

```
SmallMetro_TS_Clean$Urbanization <- "Small Metro"
SmallMetro_TS_Clean$Intent <- "Other"
SmallMetro_TS_Clean$Date <- as.yearmon(seq.Date(from = as.Date("1999-01-01"), to = as.Date("2021-12-021"), by = "month"))


###### Medium Metro ######
MediumMetro_temp <- FirearmDeaths %>% filter(Intent == "Other", Urbanization == "Medium Metro")
MediumMetro_TS <- ts(MediumMetro_temp$Deaths, start=c(1999, 1), end=c(2021, 12), frequency=12)
MediumMetro_TS_Clean <- as_tibble(na_kalman(MediumMetro_TS))
MediumMetro_TS_Clean$Urbanization <- "Medium Metro"
MediumMetro_TS_Clean$Intent <- "Other"
MediumMetro_TS_Clean$Date <- as.yearmon(seq.Date(from = as.Date("1999-01-01"), to = as.Date("2021-12-021"), by = "month"))


###### Large Fringe Metro ######
LargeFringeMetro_temp <- FirearmDeaths %>% filter(Intent == "Other", Urbanization == "Large Fringe Metro")
LargeFringeMetro_TS <- ts(LargeFringeMetro_temp$Deaths, start=c(1999, 1), end=c(2021, 12), frequency=12)
LargeFringeMetro_TS_Clean <- as_tibble(na_kalman(LargeFringeMetro_TS))
LargeFringeMetro_TS_Clean$Urbanization <- "Large Fringe Metro"
LargeFringeMetro_TS_Clean$Intent <- "Other"
LargeFringeMetro_TS_Clean$Date <- as.yearmon(seq.Date(from = as.Date("1999-01-01"), to = as.Date("2021-12-021"), by = "month"))


###### Large Central Metro ######
LargeCentralMetro_temp <- FirearmDeaths %>% filter(Intent == "Other", Urbanization == "Large Central Metro")
LargeCentralMetro_TS <- ts(LargeCentralMetro_temp$Deaths, start=c(1999, 1), end=c(2021, 12), frequency=12)
LargeCentralMetro_TS_Clean <- as_tibble(na_kalman(LargeCentralMetro_TS))
LargeCentralMetro_TS_Clean$Urbanization <- "Large Central Metro"
LargeCentralMetro_TS_Clean$Intent <- "Other"
LargeCentralMetro_TS_Clean$Date <- as.yearmon(seq.Date(from = as.Date("1999-01-01"), to = as.Date("2021-12-021"), by = "month"))



##### Recombining Imputed "Other Intent" Urbanization-stratified data #####
Other_Imputed_Temp <- bind_rows(
NonCore_TS_Clean,
Micropolitan_TS_Clean,
SmallMetro_TS_Clean,
MediumMetro_TS_Clean,
LargeFringeMetro_TS_Clean,
LargeCentralMetro_TS_Clean)

Other_Imputed_Temp$Deaths <- Other_Imputed_Temp$x
Other_Imputed_Temp$x <- NULL


##### Combing Imputed "Other Intent" Data with Homicide and Suicide Data #####
Homicide_Suicide_Data <- FirearmDeaths %>% filter(Intent != "Other")
Homicide_Suicide_Data$Date <- as.yearmon(anydate(Homicide_Suicide_Data$`Month Code`))
Homicide_Suicide_Data$`Month Code` <- NULL

Firearm <- bind_rows(Homicide_Suicide_Data,Other_Imputed_Temp); Firearm
```

© 2023 Lundstrom EW et al. *JAMA Network Open.*

```
Firearm$Date <- yearmonth(Firearm$Date)


#### Main Analysis ####

###### By Intent ######

## Creating TS
FirearmTS <- as_tsibble(Firearm, index = Date, key = c(Intent, Urbanization)); FirearmTS


## Creating Hierarchical Structure
FirearmAggTS <- FirearmTS %>% aggregate_key(Intent/Urbanization,
                                            Deaths = sum(Deaths, na.rm = TRUE)); FirearmAggTS

## Creating Aggregate Training Set (Pre-COVID Time Series: Jan 1999 - March 2020)
FirearmAggTS_PreCovid <- FirearmAggTS %>% filter_index(. ~ "2020 Mar"); FirearmAggTS_PreCovid


## Building Ensemble Model
Ensemble_Model <- FirearmAggTS_PreCovid %>%
  model(Deaths = combination_model(ARIMA(Deaths),
                                   ETS(Deaths),
                                   cmbn_args = list(weights = "inv_var"))) %>%
  reconcile(min_trace(Deaths))



## Forecasting
Death_Forecasts <- Ensemble_Model %>% forecast(h = 21) %>%
  filter(.model == "min_trace(Deaths)"); Death_Forecasts




###### By urbanization ######

## Creating TS
FirearmTS <- as_tsibble(Firearm, index = Date, key = c(Intent, Urbanization))


### Creating Hierarchical Structure
FirearmAggTS_Urbaniz <- FirearmTS %>% aggregate_key(Urbanization/Intent,
                                                    Deaths = sum(Deaths, na.rm = TRUE)); FirearmAggTS


## Creating Aggregate Training Set (Pre-COVID Time Series: Jan 1999 - March 2020)
FirearmAggTS_PreCovid_Urbaniz <- FirearmAggTS_Urbaniz %>% filter_index(. ~ "2020 Mar");
FirearmAggTS_PreCovid


## Building Ensemble Model
Ensemble_Model_Urbaniz <- FirearmAggTS_PreCovid_Urbaniz %>%
  model(Deaths = combination_model(ARIMA(Deaths),
                                   ETS(Deaths),
```

© 2023 Lundstrom EW et al. *JAMA Network Open.*

```
                                   cmbn_args = list(weights = "inv_var"))) %>%
  reconcile(min_trace(Deaths))



## Forecasting
Death_Forecasts_Urbaniz <- Ensemble_Model_Urbaniz %>% forecast(h = 21) %>%
  filter(.model == "min_trace(Deaths)"); Death_Forecasts_Urbaniz




###### Forecast Totals for COVID period ######

## Overall and by Intent
Death_Forecast_Total_CI <- Death_Forecasts %>%
  filter(.model == "min_trace(Deaths)") %>%
  as_tibble() %>%
  group_by(Intent, Urbanization) %>%
  summarise(Mean = sum(.mean),
            CI = sum(Deaths)) %>%
  mutate(`99%` = hilo(CI, 99)) %>%
  unpack_hilo('99%'); Death_Forecast_Total_CI



## By Urbanization
Death_Forecast_Total_CI_Urbaniz <- Death_Forecasts_Urbaniz %>%
  filter(.model == "min_trace(Deaths)") %>%
  as_tibble() %>%
  group_by(Urbanization, Intent) %>%
  summarise(Mean = sum(.mean),
            CI = sum(Deaths)) %>%
  mutate(`99%` = hilo(CI, 99)) %>%
  unpack_hilo('99%'); Death_Forecast_Total_CI_Urbaniz




###### Plotting Figure One ######
Death_Forecasts %>% filter(Intent == "Homicide" |
                             Intent == "Suicide",
                           is_aggregated(Urbanization),
                           .model == "min_trace(Deaths)") %>%
  autoplot(FirearmAggTS,
           level = 99) +
  geom_vline(aes(xintercept = anydate("2020 April")), linetype = 2, alpha = 0.5) +
  facet_wrap(vars(Intent), scales = "free_y", ncol = 1) +
  theme_classic() +
  theme(legend.position="none") +
  labs(x = "Year",
       y = "Firearm Deaths")
```

© 2023 Lundstrom EW et al. *JAMA Network Open.*

```
#### Model Validation - Time series cross validation (TSCV) ####

##### TAKES A LONG TIME TO RUN! #####

##### Total and Aggregated by Intent #####
### TSCV (by Intent) data
FirearmAggTS_PreCovid_cv <- FirearmAggTS_PreCovid %>%
  stretch_tsibble(.step = 1, .init = 60); FirearmAggTS_PreCovid_cv


### TSCV (by Intent) Models
TSCV_Forecasts <- FirearmAggTS_PreCovid_cv %>%
  model(ARIMA = ARIMA(Deaths),
        ETS = ETS(Deaths),
        Ensemble = combination_model(ARIMA(Deaths),
                                     ETS(Deaths),
                                     cmbn_args = list(weights = "inv_var"))) %>%
  reconcile(min_trace(ARIMA),
            min_trace(ETS),
            min_trace(Ensemble)) %>%
  forecast(h = 21); TSCV_Forecasts


### MAPE
TSCV_MAPE <- TSCV_Forecasts %>%
  filter(.model == "min_trace(ARIMA)" |
         .model == "min_trace(ETS)" |
         .model == "min_trace(Ensemble)") %>%
  accuracy(FirearmAggTS_PreCovid_cv,
           by = c(".model", "Urbanization", "Intent")); TSCV_MAPE


##### Aggregated by Urbanization #####
### TSCV (by Urbanization) data
FirearmAggTS_PreCovid_cv_Urbaniz <- FirearmAggTS_PreCovid_Urbaniz %>%
  stretch_tsibble(.step = 1, .init = 60); FirearmAggTS_PreCovid_cv_Urbaniz


### TSCV (by Urbanization) Models
TSCV_Forecasts_Urbaniz <- FirearmAggTS_PreCovid_cv_Urbaniz %>%
  model(ARIMA = ARIMA(Deaths),
        ETS = ETS(Deaths),
        Ensemble = combination_model(ARIMA(Deaths),
                                     ETS(Deaths),
                                     cmbn_args = list(weights = "inv_var"))) %>%
  reconcile(min_trace(ARIMA),
```

© 2023 Lundstrom EW et al. *JAMA Network Open.*

```
          min_trace(ETS),
          min_trace(Ensemble)) %>%
  forecast(h = 21); TSCV_Forecasts_Urbaniz



#### MAPE
TSCV_MAPE_Urbaniz <- TSCV_Forecasts_Urbaniz %>%
  filter(.model == "min_trace(ARIMA)" |
         .model == "min_trace(ETS)" |
        .model == "min_trace(Ensemble)") %>%
  accuracy(FirearmAggTS_PreCovid_cv_Urbaniz,
         by = c(".model", "Urbanization", "Intent")); TSCV_MAPE_Urbaniz
```

© 2023 Lundstrom EW et al. *JAMA Network Open.*
