# Supplementary material for: Excess US Firearm Mortality During the COVID-19 Pandemic Stratified by Intent and Urbanization
Source: JAMA Netw Open. 2023 Jul 13;6(7):e2323392. doi: 10.1001/jamanetworkopen.2023.23392 (PMC10346122; doi:10.1001/jamanetworkopen.2023.23392)
Supplement: Supplement 2. — Data Sharing Statement [file jamanetwopen-e2323392-s002.pdf]

Data Sharing Statement

Lundstrom. Excess US Firearm Mortality During the COVID-19 Pandemic Stratified by Intent and Urbanization. *JAMA Netw Open*. Published July 13, 2023.
doi:10.1001/jamanetworkopen.2023.23392

Data
Data available: Yes
Data types: Data (not involving human participants)
How to access data: EWL had full access to all the data in the study and takes responsibility for the integrity of the data and the accuracy of the data analysis. The data is available at ewlundstrom@gmail.com upon reasonable request.
When available: With publication

Supporting Documents
Document types: Statistical/analytic code
How to access documents: R code available at the end of the Supplementary File - Detailed Methodology document.
When available: With publication

Additional Information
Who can access the data: Anyone requesting to download the data.
Types of analyses: Any.
Mechanisms of data availability: Email to ewlundstrom@gmail.com upon reasonable request.
